# MRI-based automatic identification and segmentation of extrahepatic cholangiocarcinoma using deep learning network

**DOI:** 10.1186/s12885-023-11575-x

**Published:** 2023-11-10

**Authors:** Chunmei Yang, Qin Zhou, Mingdong Li, Lulu Xu, Yanyan Zeng, Jiong Liu, Ying Wei, Feng Shi, Jing Chen, Pinxiong Li, Yue Shu, Lu Yang, Jian Shu

**Affiliations:** 1 Department of Radiology, The Affiliated Hospital of Southwest Medical University, Luzhou, Sichuan 646000 China; 2 Department of Research and Development, Shanghai United Imaging Intelligence Co., Ltd., Shanghai, China; 3 Department of Oncology, The Affiliated Hospital of Southwest Medical University, Luzhou, Sichuan 646000 China

**Keywords:** Extrahepatic cholangiocarcinoma, Magnetic resonance imaging, Automatic identification, Automatic segmentation, Deep learning

## Abstract

**Background:**

Accurate identification of extrahepatic cholangiocarcinoma (ECC) from an image is challenging because of the small size and complex background structure. Therefore, considering the limitation of manual delineation, it’s necessary to develop automated identification and segmentation methods for ECC. The aim of this study was to develop a deep learning approach for automatic identification and segmentation of ECC using MRI.

**Methods:**

We recruited 137 ECC patients from our hospital as the main dataset (C1) and an additional 40 patients from other hospitals as the external validation set (C2). All patients underwent axial T1-weighted imaging (T1WI), T2-weighted imaging (T2WI), and diffusion-weighted imaging (DWI). Manual delineations were performed and served as the ground truth. Next, we used 3D VB-Net to establish single-mode automatic identification and segmentation models based on T1WI (model 1), T2WI (model 2), and DWI (model 3) in the training cohort (80% of C1), and compared them with the combined model (model 4). Subsequently, the generalization capability of the best models was evaluated using the testing set (20% of C1) and the external validation set (C2). Finally, the performance of the developed models was further evaluated.

**Results:**

Model 3 showed the best identification performance in the training, testing, and external validation cohorts with success rates of 0.980, 0.786, and 0.725, respectively. Furthermore, model 3 yielded an average Dice similarity coefficient (DSC) of 0.922, 0.495, and 0.466 to segment ECC automatically in the training, testing, and external validation cohorts, respectively.

**Conclusion:**

The DWI-based model performed better in automatically identifying and segmenting ECC compared to T1WI and T2WI, which may guide clinical decisions and help determine prognosis.

**Supplementary Information:**

The online version contains supplementary material available at 10.1186/s12885-023-11575-x.

## Introduction

Cholangiocarcinoma (CCA) is one of the most aggressive human malignant tumors, arising from the biliary epithelium and peribiliary glands [1, 2]. CCA is categorized into both intrahepatic and extrahepatic forms (ECC); ECC arises in the bile ducts outside the liver parenchyma and accounts for approximately 80% of all CCA [3, 4]. The incidence and mortality rates of ECC have increased gradually over the last decade, and its prognosis remains poor [3]. Surgical resection is the most effective therapeutic approach for ECC treatment. It is usually difficult for inexperienced radiologists to accurately identify ECC lesions from images because of the small volume and complex background structure. A clear and accurate boundary is important for volume assessment, tumor identification/segmentation, and even effective treatment, such as surgical therapy and local radiotherapy; it can help in making the appropriate clinical decisions and reduce margin-positive resection or irradiation [5, 6]. Meanwhile, with the development of artificial intelligence in radiology, precise identification and segmentation of tumor are also required for further analysis.

Currently, a variety of noninvasive, economic, and repeatable medical imaging technologies, including ultrasonography [7], computed tomography [8], positron emission tomography [9] and magnetic resonance imaging (MRI) [10, 11], can improve the accuracy of ECC diagnosis. MRI is considered the most accurate and least invasive modality for detecting ECC due to its superior soft tissue contrast, which is essential for tumor staging and edge delineation. Another benefit of MRI is the ability to obtain images reflecting functional tissue information, such as diffusion-weighted imaging (DWI) that can visualize the microscopic thermal motion of water molecules in tissue [12, 13]. Hence, precise MRI-based tumor identification and segmentation are desirable. However, the delineation process was completed manually on multi-slice images, which is subjective, labor-consuming, and time-consuming. Moreover, manual segmentation is prone to error, has high intra- and inter-operator variability, and greatly depends on the skills of the physician or doctor who performs the segmentation task [14]. Considering all the above problems, an automatic and fast tumor identification and segmentation technique, which helps in the treatment and surgical planning of ECC, is urgently needed to assist intelligent medicine.

Deep learning has achieved great success in medical imaging due to their impressive automatic segmentation performance. With the development of deep learning, artificial neural network-based techniques have been used to tackle segmentation tasks in different diseases or organs, such as brain tumor [15–17], lung cancer [18], breast tumor [19], hepatocellular carcinoma [20], cervical cancer [21], gastric tumor [22], rectal cancer [23], liver [24], and pancreas [25]. Currently, most researchers of CCA image processing techniques still segment the liver and tumor manually to achieve accuracy [26–30]. However, as mentioned previously, this method is time-consuming, has strong subjectivity and poor repeatability, and makes it difficult to realize 3D segmentation. In contrast, automatic methods have the potential to save time and decrease inter-observer variations. Because of the heterogeneity of bile duct tissue and the similar density/intensity of various structures, there is no general segmentation algorithm with high recognition. Therefore, a specific, automatic, and high-precision image segmentation algorithm is the development trend of the future. To the best of our knowledge, there is a paucity of deep learning algorithms for automatic identification and segmentation of ECC.

Therefore, the aim of this study was to investigate the performance of an MRI-based deep learning algorithm for automatic identification and segmentation of ECC.

## Materials and methods

### Patients

**Fig. 1:**
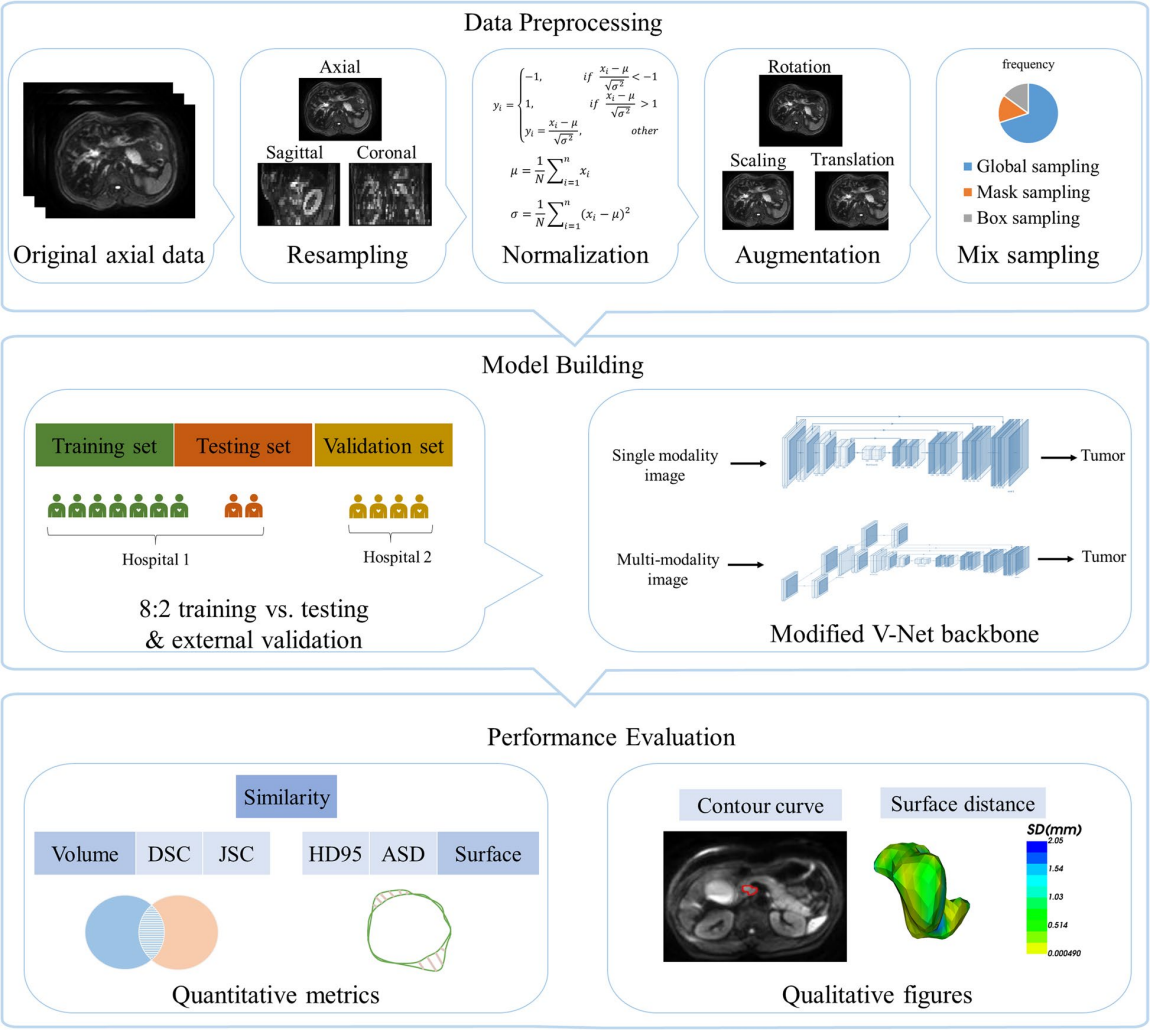
The overview of the experimental procedure

All patients met the inclusion and exclusion criteria (Supplementary material S1). Finally, 177 patients with a pathological diagnosis of ECC between January 2011 and December 2021 were included in our analysis. Of these patients, 137 (cohort 1) were from our hospital and the remaining 40 (cohort 2) were from other hospitals. Cohort 1 was randomly divided into training (*n* = 109) and testing sets (*n* = 28) at a ratio of 8:2. Cohort 2 (*n* = 40) was included as the external validation cohort. All patients experienced preoperative MRI scanning. The detailed MRI protocols were listed in Supplementary material S2. Besides, some clinical and pathological characteristics of patients were collected. Figure 1 presents a general overview of the experimental procedure.

### Automatic segmentation model construction

#### Automatic tumor segmentation

First, manual delineations were performed and served as the ground truth in our study. The details were showed in Supplementary material S3. For automatic segmentation of ECC, image preprocessing and data augmentation was performed using Python 3.7 (Supplementary material S4). Next, we adopted a 3D VB-Net as the backbone in the proposed framework. VB-Net is a modified network that combines V-Net with bottleneck modules to reduce and combine feature-map channels, which encourages much smoother gradient flow and shows easier optimization/convergence [31]. First, we randomly selected 80% of the samples (*n* = 109) as the training set and the remaining 20% as the test set (*n* = 28) from cohort 1. Then, using the VB-Net algorithm, single-modality models were trained using T1WI (model 1), T2WI (model 2), and DWI (model 3) respectively.

**Fig. 2:**
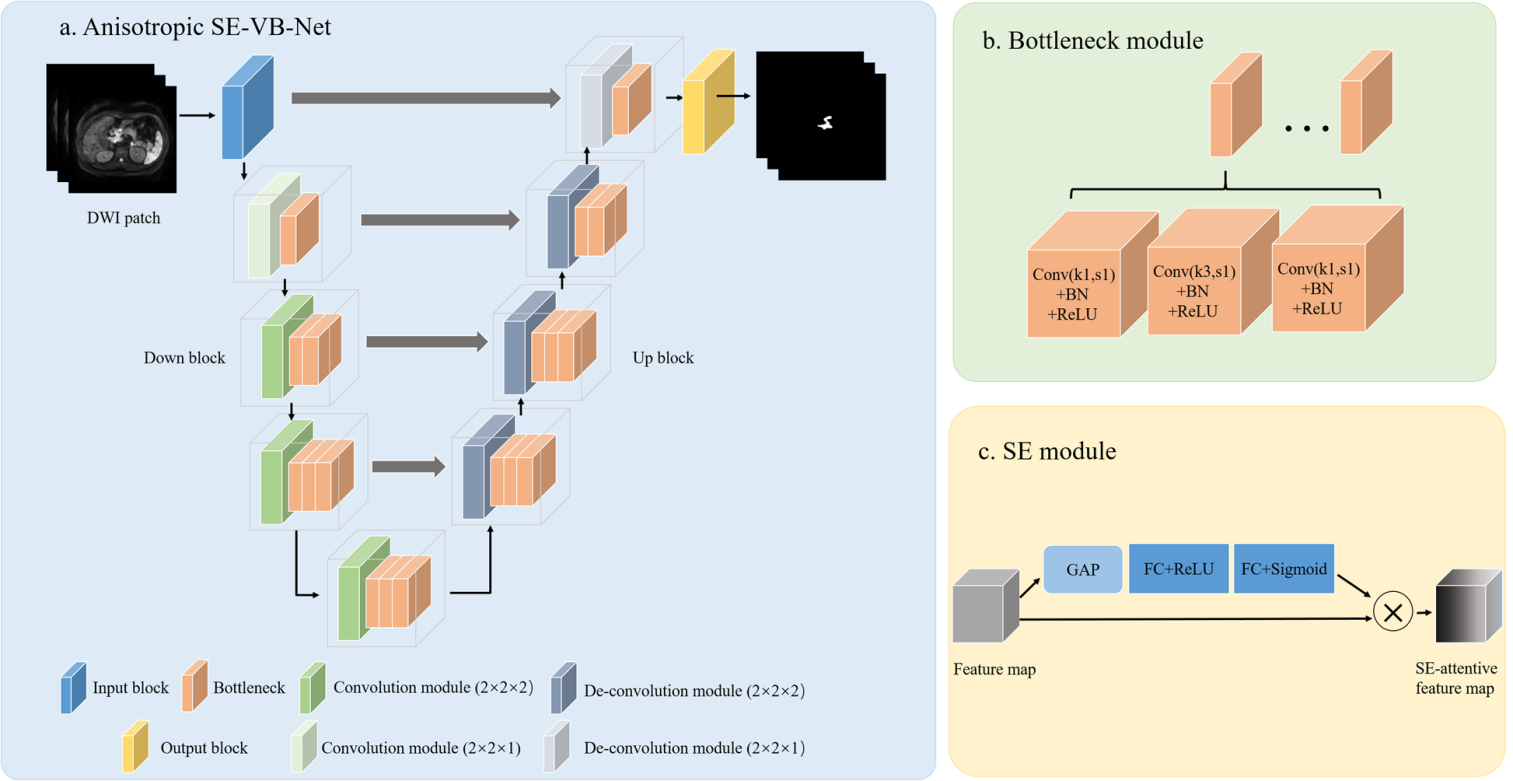
The architecture of the VB-Net algorithm for single-modality models. The VB-Net included one input block, four down blocks, four up blocks, and one output block (**a**). The bottleneck module consisted a certain number of bottleneck structures (**b**). **c** shows the squeeze-and-excitation (SE) module. GAP, global averaging pooling layer; FC, fully connected layer; BN, batch normalization layer. Conv (k1,s1): convolution layer whose kernel size and stride size was set as 1 × 1 × 1and 1 × 1 × 1, respectively. Conv(k3,s1): convolution layer whose kernel size and stride size was set as 3 × 3 × 3and 1 × 1 × 1, respectively

As illustrated in Fig. 2a, the VB-Net included one input block, four down blocks, four up blocks, and one output block. The input block consisted of one convolution module, and the output block consisted of a convolution module, global average pooling layer, and Softmax layer. Specifically, the down/up block comprised one convolution/de-convolution module, one bottleneck module, and a squeeze-and-excitation (SE) module [2, 32]. Figure 2a showed the specific number of bottle structures in the bottle module of the four down blocks, which was set as 1, 2, 3, and 3. The specific number of bottle structures in the bottle module of the four upper blocks was set as 3, 3, 2, and 1.

Figure 2b showed the bottleneck module consisted of a certain number of bottleneck structures, which included one convolution module (kernel size was set as 1 × 1 × 1, stride size was set as 1 × 1 × 1, followed by one batch normalization (BN) layer and one rectified linear unit (ReLU) layer) to reduce the channel of the feature maps and another two convolution modules (kernel size was set as 3 × 3 × 3 and 1 × 1 × 1; stride size was set as 1 × 1 × 1 and 1 × 1 × 1, respectively; followed by one BN layer and one ReLU layer) to restore the initial channels of the feature maps. A bottleneck module was combined in the network to reduce the number of network parameters and thereby speed up network convergence. In our network architecture, both down and up blocks took the form of a residual SE structure (Fig. 2c). Supplementary material S5 described the details of SE module.

**Fig. 3:**
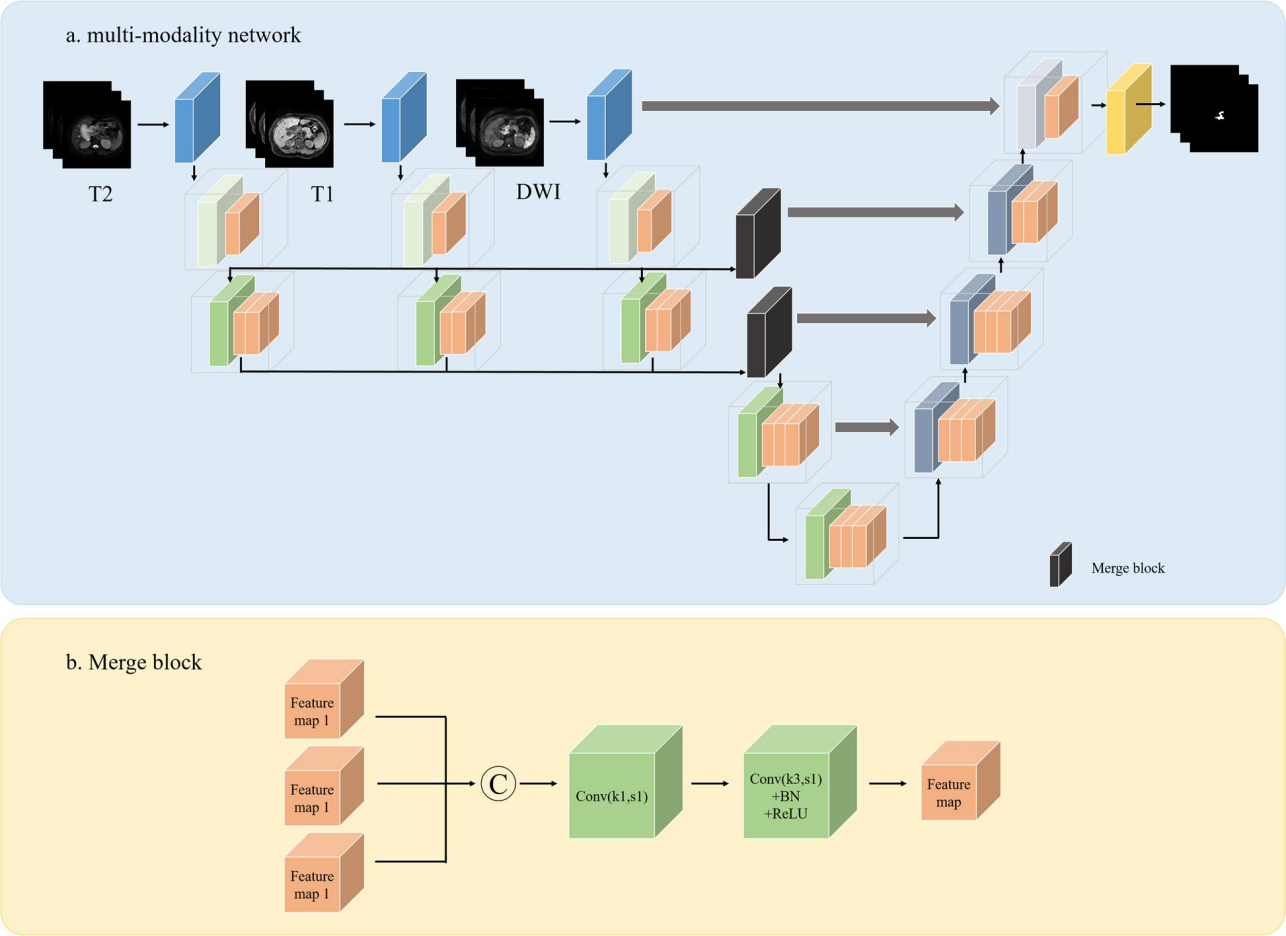
The architecture of the VB-Net algorithm for multi-modality models

Furthermore, the combined segmentation model (model 4) was also trained using the combination of T1WI, T2WI, and DWI, and compared with the above mentioned single-modality models. The major operation was similar to the processes of models 1, 2, and 3. Figure 3 showed the detailed architecture. The differences between the single-modality and combined network were described in Supplementary material S6.

#### Loss functions and validation of segmentation model

In all training processes, the loss functions of dice loss, focal loss, and soft dice loss were combined to optimize the model (Supplementary material S7). Then, the parameter combinations that resulted in the best training models were chosen for subsequent analysis. The generalization capability of the best models (including models 1, 2, 3, and 4) was evaluated using an independent testing set (20% cohort 1, *n* = 28) and an external validation set (cohort 2, *n* = 40). All the procedures for automatic tumor segmentation were implemented in Python 3.7 and PyTorch 1.7.0, with one NVIDIA Tesla V100 graphics processing unit. In our method, the Adam optimizer (initial learning rate = 0.0001) algorithm was chosen to minimize the loss of neural network and the batch size was set as 16. The training process was considered to have converged if the loss stopped decreasing for 20 epochs, and the optimal training epoch of model was selected based on the metric of DSC in the testing dataset.

#### Evaluation metrics for segmentation

To evaluate the accuracy of the segmentation algorithm, the results of the automatically segmented data were compared with those of the ground truth, using both volumetric and surface analysis statistics. Evaluation metrics (Supplementary material S8), including the Dice similarity coefficient (DSC), 95th percentile of Hausdorff distance (HD95), average surface distance (ASD), and Jaccard similarity coefficient (JSC), were calculated using python3.7. Besides, we evaluated the success rate of identification and segmentation for each model, indicating its ability to detect coarse tumor locations.

Furthermore, Mann–Whitney U test was used to compare the differences in the above metrics (DSC, HD95, ASD, and JSC) between model 3 and the other models in the training, testing, and validation groups.

### The application of automatic segmentation model

In order to further validate the automatic identification and segmentation ability of our constructed model, 30 normal participants (group 1), 30 subjects with extrahepatic bile duct stones (group 2), and 28 ECC patients in the testing cohort (group 3) were included in our study. Abdominal MRI images (axial T1WI, T2WI, and DWI) of all patients in these three groups were imported into our 3D anisotropic SE-VB-Net, namely Ani-SE-VB-Net, to observe automatic identification and segmentation of extrahepatic bile duct region. The success rate and DSC were calculated to further evaluate the performance of the proposed model.

## Results

### Patients

The study sample consisted of 99 females and 78 males with an age of 61.0 (55.0, 67.0) years, ranging from 28 to 87 years. All the tumors were confirmed as adenocarcinomas and were divided into well-differentiated (*n* = 64), moderately differentiated (*n* = 81), and poorly differentiated (*n* = 32) groups. And 49 subjects were diagnosed with lymphatic metastasis by pathological examination. The detailed patient characteristics are summarized in Table 1.

**Table 1:** Clinical and pathological characteristics of patients with ECC

Characteristic	Training cohort	Testing cohort	Validation cohort
Patients number	109	28	40
Gender			
Male	65 (59.6%)	15 (53.6%)	19 (47.5%)
Female	44 (40.4%)	13 (46.4%)	21 (52.5%)
Age (year)	61.0 (53.5, 66.0)	60.0 (50.3, 66.8)	64.0 (58.0, 71.8)
Location			
Perihilar	57 (52.3%)	14 (50.0%)	21 (52.5%)
Distal	52 (47.7%)	14 (50.0%)	19 (47.5%)
Lesion size (cm)	1.2 (0.9, 1.5)	0.9 (0.7, 1.3)	1.0 (0.8, 1.4)
Differentiation degree			
Well	41 (37.6%)	11 (39.3%)	12 (30.0%)
Moderately	52 (47.7%)	9 (32.1%)	20 (50.0%)
Poorly	16 (14.7%)	8 (28.6%)	8 (20.0%)
Lymphatic status			
Positive	31 (28.4%)	7 (25.0%)	11 (27.5%)
Negative	78 (71.6%)	21 (75.0%)	29 (72.5%)

Lesion size was defined as the maximum diameter on transverse images

The values of age and lesion size were expressed as median (interquartile range) due to abnormal distribution

### Automatic segmentation model construction

In this study, automatic identification and segmentation models for ECC were successfully developed using an Ani-SE-VB-Net. The DWI-based model showed the best identification ability in the training, testing, and external validation cohorts, with success rates of 0.980, 0.786, and 0.725, respectively. Furthermore, it yielded an average DSC of 0.922, 0.495, and 0.466 for segmenting ECC automatically in the training, testing, and external validation cohorts, respectively. In the training set, the other models, including model 1, model 2 and model 4, also yielded high success rates of 0.961, 0.963, and 1.000, respectively, in automatically identifying tumor lesions. The average DSC values of models 1, 2, and 4 were 0.753, 0.826, and 0.775, respectively.

For the testing cohort, model 3, based on DWI, showed an average HD95 of 5.464, ASD of 1.431, and JSC of 0.360. The combined model (model 4) yielded a success rate of 0.786, with an average DSC of 0.462, an average HD95 of 6.834, an average ASD of 2.922, and an average JSC of 0.331, which were superior to those of models 1 and 2. However, there were 9, 8, and 6 lesions on T1WI, T2WI, and DWI, respectively that were not identified, with a DSC of 0 in the testing set. Among them, 4 lesions were too small (diameter less than 7.0 mm) to be identified. The remaining cases had unclear boundaries and showed isointensity on T1WI/T2WI/DWI with adjacent tissues.

For the validation cohort, the DWI-based model displayed an average HD95 of 6.767, ASD of 2.394, and JSC of 0.332. However, 11 lesions were unidentifiable on DWI due to small volume or obscure boundary caused by the isointensity of the tumor in this cohort. Nevertheless, a satisfactory result was obtained in our study, in which the identification ability of the DWI-based model reached 1.000 in the training, testing, and validation groups when lesions with isointensity on DWI or small size (diameter less than 7.0 mm) were excluded. Further details are provided in Table 2.

**Table 2:** The performance of the single-modality and combined model for automatic segmentation of ECC

Data	Modality	Success rate	DSC	HD95	ASD	JSC
Training	Model 1	0.961	0.753 $\pm$ 0.103	1.886 $\pm$ 1.363	0.609 $\pm$ 0.371	0.614 $\pm$ 0.120
	Model 2	0.963	0.826 ± 0.099	3.689 ± 9.858	0.906 ± 3.248	0.714 ± 0.126
	Model 3	0.980	0.922 $\pm$ 0.055	0.720 $\pm$ 0.488	0.134 $\pm$ 0.104	0.860 $\pm$ 0.087
	Model 4	1.000	0.775 ± 0.185	4.641 ± 6.777	1.516 ± 2.744	0.662 ± 0.201
Testing	Model 1	0.679	0.287 $\pm$ 0.223	10.848 $\pm$ 7.414	4.141 $\pm$ 3.600	0.190 $\pm$ 0.174
	Model 2	0.714	0.249 $\pm$ 0.221	15.626 $\pm$ 10.976	5.018 $\pm$ 6.660	0.165 $\pm$ 0.180
	Model 3	0.786	0.495 $\pm$ 0.227	5.464 $\pm$ 3.994	1.431 $\pm$ 1.413	0.360 $\pm$ 0.210
	Model 4	0.786	0.462 $\pm$ 0.245	6.834 $\pm$ 7.385	2.922 $\pm$ 5.079	0.331 $\pm$ 0.221
Validation	Model 1	0.375	0.229 $\pm$ 0.182	13.854 $\pm$ 9.951	5.084 $\pm$ 4.921	0.142 $\pm$ 0.124
	Model 2	0.300	0.222 $\pm$ 0.159	8.712 $\pm$ 6.934	3.167 $\pm$ 3.291	0.135 $\pm$ 0.108
	Model 3	0.725	0.466 $\pm$ 0.232	6.767 $\pm$ 6.634	2.394 $\pm$ 3.704	0.332 $\pm$ 0.191
	Model 4	0.500	0.341 $\pm$ 0.220	27.364 $\pm$ 40.19	17.448 $\pm$ 28.698	0.229 $\pm$ 0.184

Model 1, model 2 and model 3 were constructed based on T1WI, T2WI and DWI respectively. Model 4 was developed based on the combination of the three sequence

*DSC* Dice similarity coefficient; HD95, 95% Hausdorff distance, *ASD* Average symmetric surface distance, *JSC* Jaccard similarity coefficient. All metrics were expressed as mean ± SD

**Fig. 4:**
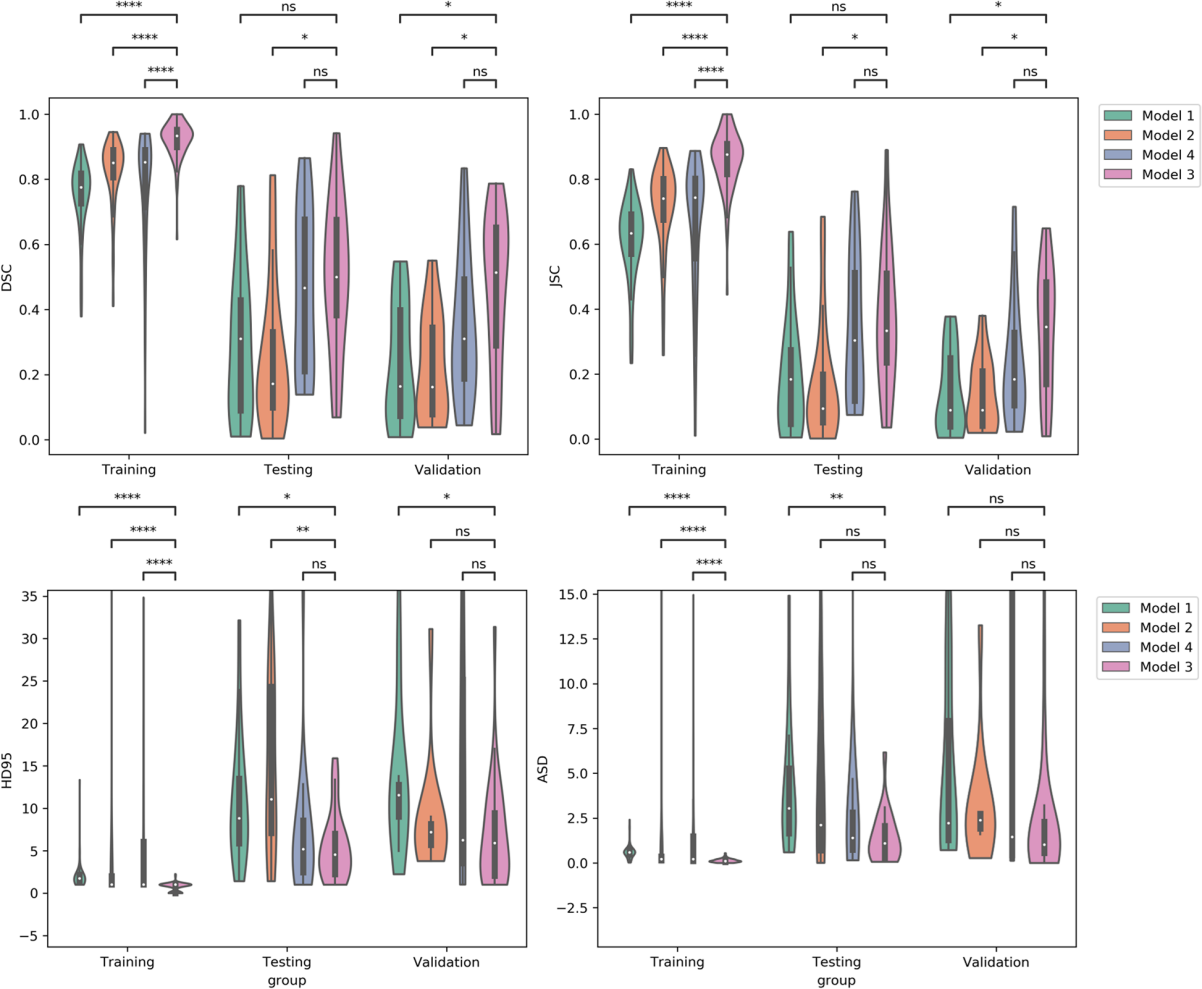
The violin distributed diagram of four metrics in training, testing and validation dataset. Models 1, 2, and 3 were constructed based on T1WI, T2WI, and DWI respectively. Model 4 was developed based on the combination of the three sequence. DSC, Dice similarity coefficient; HD95, 95% Hausdorff distance; ASD, average symmetric surface distance; JSC, Jaccard similarity coefficient. ns: 0.05 < *p* ≤ 1.00; *: 0.01 < *p* < 0.05; **: 0.001 < *p* ≤ 0.01; ***: 0.0001 < *p* ≤ 0.001; ****: *p* ≤ 0.0001

Figure 4 shows the violin distributed diagram of the four metrics (DSC, HD95, ASD, and JSC) in the training, testing, and validation datasets. As shown in Fig. 4, all metrics were significantly different between model 3 and the other models (including models 1, 2, and 4) (*p* < 0.05) in the training dataset. In the testing dataset, the differences in HD95 (*p* < 0.05) and ASD (*p* ≤ 0.01) between models 3 and 1 were significant. In addition, there were significant differences in HD95 (*p* ≤ 0.01), DSC (*p* < 0.05), and JSC (*p* < 0.05) between models 3 and 2. In the external validation dataset, DSC, JSC, and HD95 were significantly different between models 3 and 1 (*p* < 0.05), and the differences in DSC and JSC (*p* < 0.05) between models 3 and 2 were also significant.

**Fig. 5:**
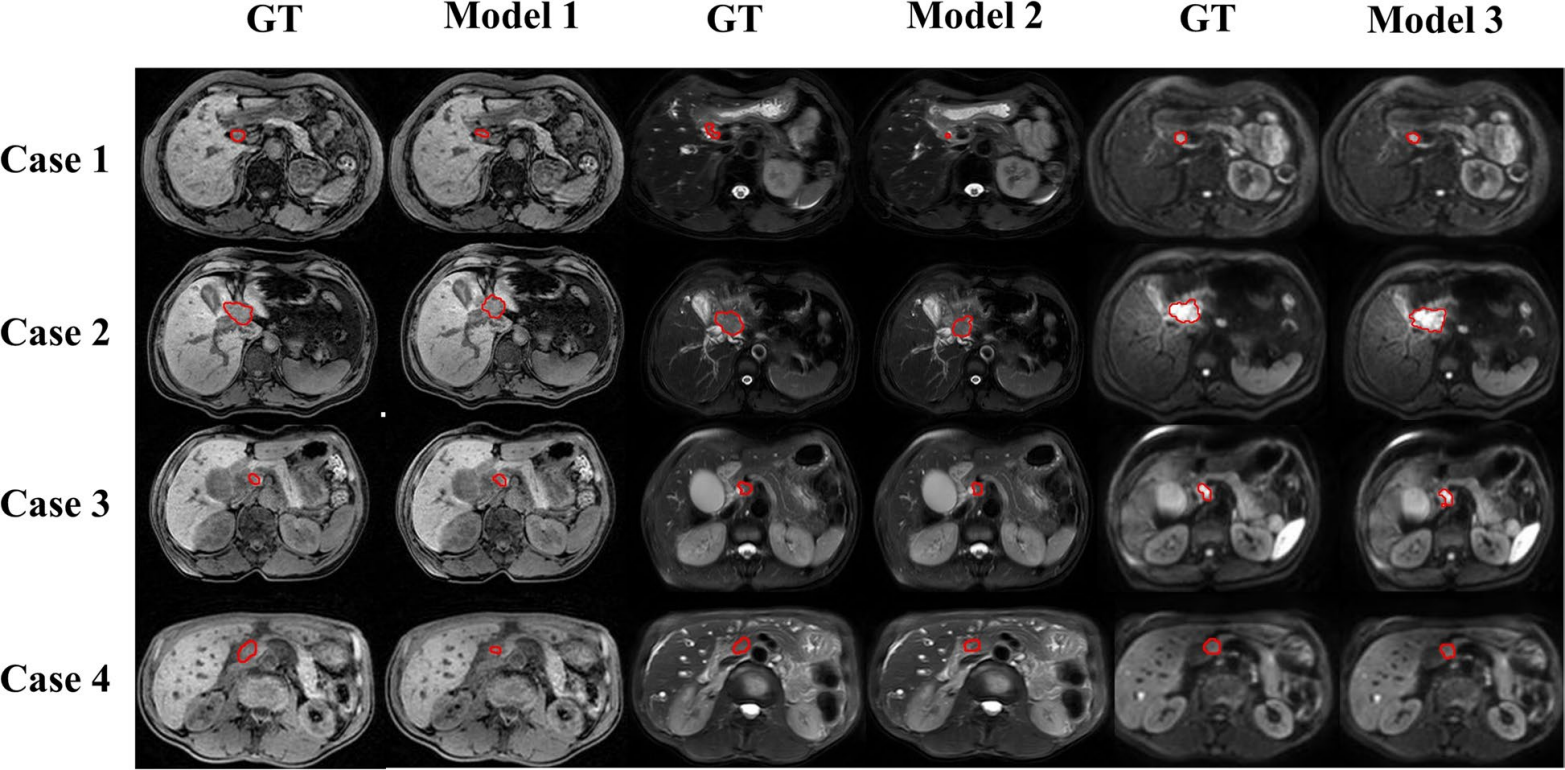
2D visualization of the ground truths and segmented slices using different models. Models 1, 2, and 3 were constructed based on T1WI, T2WI, and DWI respectively. GT, ground truth

**Fig. 6:**
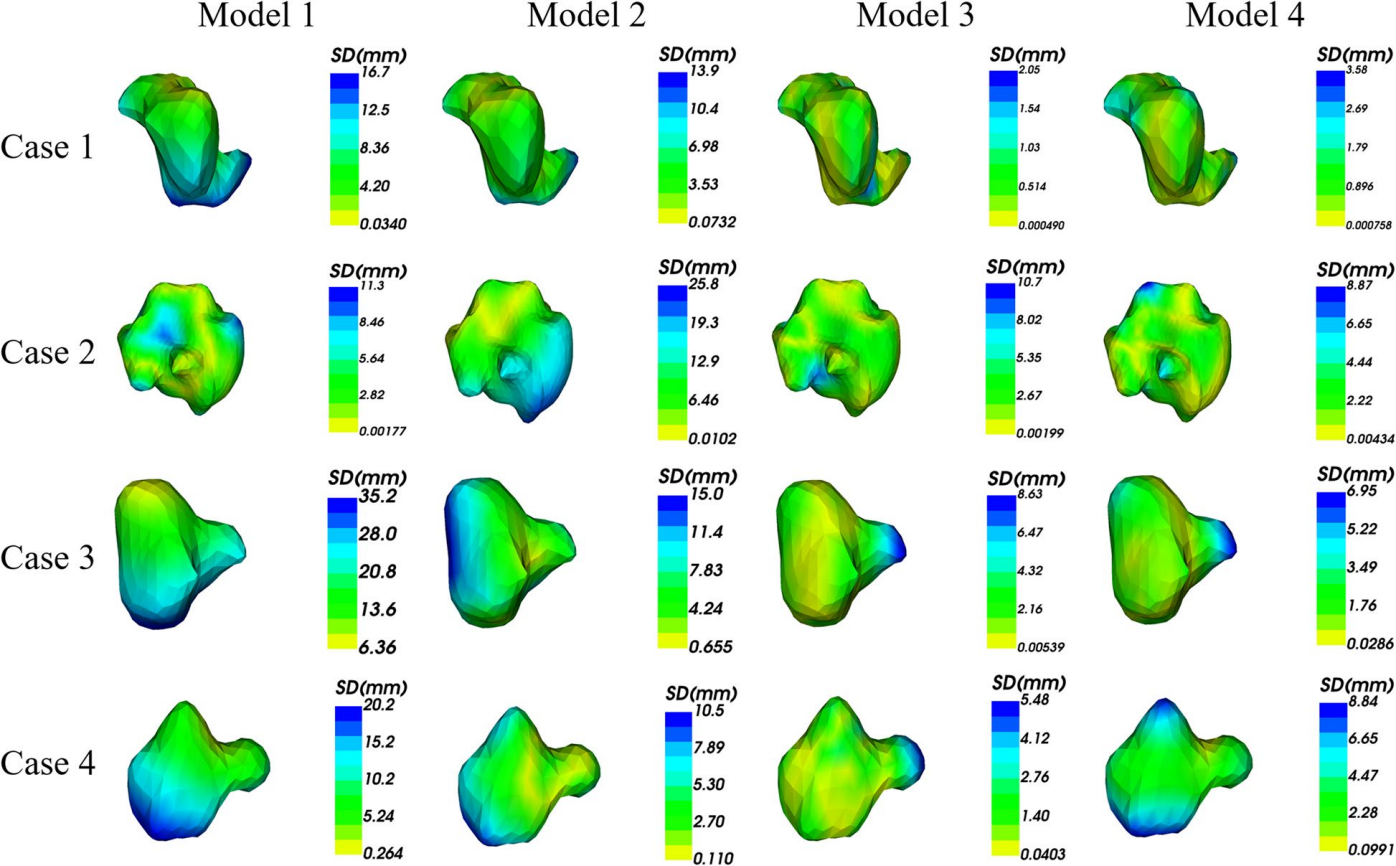
3D visualization of the surface distance between segmented results and ground truths with different colors representing different surface distances. Models 1, 2, 3 and 4 were constructed based on T1WI, T2WI, DWI and combined sequences respectively. GT, ground truth

In addition, Fig. 5 shows the 2D visualization of the ground truths and the prediction of tumor boundaries using different models. Our method had segmented boundaries similar to ground truths. Figure 6 displays the 3D visualization of the surface distance between the segmented results and ground truths, with different colors representing different surface distances. We mapped the ground truths to the corresponding prediction volume of each model, and this visualization made the comparison more intuitive.

### The application of automatic segmentation model

For groups 1 and 2, all patients experienced failed identification and segmentation. However, for group 3, the DWI-based model still showed the best segmentation ability with a success rate of 0.786 and a DSC of 0.495 compared to other models.

## Discussion

In our study, we developed the first MRI-based automatic identification and segmentation model for ECC. A 3D Ani-SE-VB-Net algorithm was used for automatic identification and segmentation of ECC in T1WI, T2WI, and DWI sequences. We used a large-scale data augmentation scheme to mitigate the limited size of our dataset. Our novel approach has not been previously explored for ECC with a small size and complex background structure, which is beneficial to our patient sample. This suggests that the clinical use of 3D Ani-SE-VB-Net algorithm is promising in terms of automatic identification and segmentation of ECC. Therefore, it may also be beneficial for the selection of treatment strategies and for improving the prognosis of patients with ECC.

Currently, there are some difficulties in the early detection of ECC and identification of benign and malignant bile duct dilatation. From the analysis presented in this work, the DWI-based model yielded a success rate of more than 70% in identifying tumors in both the testing and external validation cohorts, which suggests that Ani-SE-VB-Net has great advantages in the exploration of ECC. Moreover, the automatic identification ability of deep learning reached 100% for lesions with hyperintensity on DWI and a diameter ≥ 7.0 mm, in our training, testing, and validation cohorts. Our previous study demonstrated that more than 95% of ECC showed hyperintensity on DWI [11]. Hence, our model has a better ability to automatically identify ECC from abdominal MRI. Using our algorithm to automatically identify the presence of tumor lesions can not only help inexperienced radiologists diagnose diseases, but also help experienced experts shorten the reading time and improve diagnostic accuracy. Therefore, Ani-SE-VB-Net may be a powerful tool for radiologists to automatically identify and diagnose ECC and can guide optimal treatment planning by clinicians.

As we all know, automated delineation of tumor is an essential preliminary step for imaging-based tumor analysis and monitoring treatment outcome. These automated methods provide objective assessments of the subjectivity and intra- and inter-observer variability of manual measurements and reduce manual effort and time, which may be useful for qualitative and quantitative medical image analyses and computer-aided decision support systems. Currently, automatic segmentation techniques have been used for different diseases, especially tumors, e.g. brain tumor, lung cancer, breast tumor, and rectal cancer. For CCA, there are a few analyses that need to be explored for automatic segmentation. Selvathi et al. proposed the Fuzzy C-Means algorithm to segment liver tumors, including CCA [33]. In addition, a recent study indicated that volumetric computed tomography (CT) texture analysis using fully automatic segmentation could be utilized as a prognostic marker in patients with intrahepatic mass-forming cholangiocarcinoma, with comparable reproducibility in significantly less time compared to semi-automatic segmentation [34]. However, these immature automatic segmentations have not been introduced in detail and are unable to meet clinical needs. Furthermore, the above mentioned segmentation algorithms were developed based on intrahepatic cholangiocarcinomas. Currently, no relevant studies have separately reported automatic segmentation algorithms of ECC. It is difficult to automatically segment ECC because of its small size and complex background structure compared to intrahepatic cholangiocarcinoma. Therefore, considering the differences between ECC and intrahepatic cholangiocarcinoma, a 3D Ani-SE-VB-Net based on MRI was built and validated to automatically identify and segment ECC for the first time in our study.

In this study, we found that the constructed model demonstrated better performance and reliability in the training, testing, and validation cohorts. We established three single-mode automatic segmentation models based on T1WI, T2WI, and DWI and compared them with the combined model using three sequences. Our results indicated that the performance of the DWI-based model was much better than that of other models based on T1WI, T2WI, and combined sequences.We speculated that the inferior recognition performance observed in both T1WI and T2WI could be attributed to resemblance in texture and intensity features between the target tissue and its adjacent structures. In contrast, the majority of ECC showed hyperintensity on DWI, which was clearly distinguishable from the background tissue [11]. The prominence of lesions on DWI may be beneficial to the identification and delineation of tumors, which gives a better performance of the segmentation model in DWI compared to T1WI and T2WI. Consequently, T1WI and T2WI provided little effective and complementary information to improve segmentation performance for the combined model. Furthermore, considering the lower registration accuracy of small ECC in comparison to larger lesions or organs, the input-wise fusion of T1WI, T2WI and DWI may result in some misplaced tumor information, thereby reducing performance of model. Further analysis demonstrated that our model could successfully identify and segment ECC from MRI images mixed with those of extrahepatic bile duct stones and normal subjects. This suggests that our 3D Ani-SE-VB-Net showed excellent performance in automatically identifying and segmenting ECC by analyzing the differences in MR signals of different lesions and the heterogeneity of intralesional texture.

Of course, some cases also experienced failed segmentation, with a DSC of 0 in our study. A potential reason for this could be that some lesions were too small (diameter less than 7.0 mm), which made it difficult to delineate the boundary accurately. Another reason may be that some lesions had unclear boundaries and showed similar signals (especially some lesions exhibiting isointensity on DWI) with adjacent tissues, so that the tumor was unidentified or adjacent tissues were included. Huang et al. reported that only a small part of ECC (less than 5%) exhibited iso or hypointensity on DWI. Therefore, a DWI-based deep learning model can show an excellent ability to automatically identify and segment ECC in most cases [11]. What this suggests is that our automatic segmentation model still has a certain degree of clinical guidance.

Our study had some limitations. First, our study was retrospective and the segmentation algorithm was applied on a limited dataset involving ECC only. Therefore, prospective studies with a considerably large number of datasets are needed to further validate the robustness of our segmentation model and to make the segmentation algorithm more sensitive to smaller tumors. Second, the described segmentation algorithm was limited to axial images on T1WI, T2WI, and DWI sequences. However, other sequences such as T1-weighted dynamic contrast-enhanced images (T1-DCEI) were not included in the present study. T1-DCEI could provide a smaller layer thickness and make the lesion significantly different from the background tissue, which may facilitate tumor recognition and segmentation. We plan to design a segmentation algorithm that incorporates DWI and T1-DCEI to expand the indications for using our segmentation model. Finally, Ani-SE-VB-Net was selected and applied to our automated segmentation. However, there may be other methods suitable for this purpose that are yet to be considered, e.g. a multi-scale cascaded convolutional network. This framework consisted of three components: the multi-scale detection network (feature pyramid network), the cascade network and the classification network. These components worked together to maintain high sensitivity and eliminate false positives. Therefore, other available algorithms will be further tried to develop more accurate model in the future.

## Conclusion

In conclusion, we present the first 3D model for automated identification and segmentation of ECC with Ani-SE-VB-Net, which demonstrated enormous potential in the identification and segmentation of tumors with small sizes and complex background structures such as ECC. It should be noted that varying intensity patterns, ill-defined boundaries, and small volumes are some challenges in labeling tumors. Therefore, using the model on unseen cohorts requires caution and one cannot expect the same performance level.

### Supplementary Information

**Additional file 1:** **Supplementary material S1.** The inclusion and exclusion criteria. **Supplementary material S2.** Magnetic resonance imaging protocol. **Supplementary material S3.** Manual segmentation. **Supplementary material S4.** Image preprocessing. Data augmentation. **Supplementary material S5.Supplementary material S6.Supplementary material S7.** Loss functions. **Supplementary material S8.** Evaluation metrics for segmentation.

## Data Availability

The datasets generated and/or analysed during the current study are not publicly available due confidential information but are available from the corresponding author on reasonable request.

## Abbreviations

CCA: Cholangiocarcinoma
ECC: Extrahepatic cholangiocarcinoma
DWI: Diffusion weighted imaging
T1WI: T1-weighted imaging
T2WI: T2-weighted imaging
ROI: Region of interest
DSC: Dice similarity coefficient
HD95: The 95th percentile of Hausdorff distance
ASD: Average surface distance
JSC: Jaccard similarity coefficient
