# Supplementary material for: MRI-based automatic identification and segmentation of extrahepatic cholangiocarcinoma using deep learning network
Source: BMC Cancer. 2023 Nov 10;23:1089. doi: 10.1186/s12885-023-11575-x (PMC10636947; doi:10.1186/s12885-023-11575-x)
Supplement: Supplementary file 1 — Additional file 1: Supplementary material S1. The inclusion and exclusion criteria. Supplementary material S2. Magnetic resonance imaging protocol. Supplementary material S3. Manual segmentation. Supplementary material S4. Image preprocessing. Data augmentation. Supplementary material S5.Supplementary material S6.Supplementary material S7. Loss functions. Supplementary material S8. Evaluation metrics for segmentation. [file 12885_2023_11575_MOESM1_ESM.docx]

***Supplementary material***

**Title: MRI-based automatic identification and segmentation of extrahepatic cholangiocarcinoma using deep learning network**

**Supplementary material S1**

**The inclusion and exclusion criteria**

All patients met the following inclusion and exclusion criteria. First, all ECC patients had abdominal MRI images taken less than 2 weeks before surgical resection and had obtained pathological confirmation. Second, patients were excluded for any of the following criteria: (1) poor quality MRI images and (2) lesions with diameters less than 5 mm.

**Supplementary material S2**

**Magnetic resonance imaging** **protocol**

For cohort 1, preoperative MRI scanning was performed using a 3.0 T MRI (Achieva 3.0 T, Philips, Amsterdam, Netherlands) via a 16-channel abdominal coil. The MRI scanning range extended from the top of the diaphragm to the horizontal portion of the duodenum. The MRI sequences mainly included axial T1 high resolution isotropic volume examination sequence (T1WI), axial fat-suppressed turbo spin-echo T2-weighted imaging (T2WI), coronal turbo spin-echo T2WI sequence, axial dual-echo T1-weighted imaging breath-hold gradient-echo sequence for acquisition of in-phase and out-of-phase images, axial DWI, magnetic resonance cholangiopancreatography (MRCP), and axial T1-weighted dynamic contrast-enhanced MR images. For cohort 2, all patients underwent a 1.5/3.0T abdominal MRI scan (Philips Healthcare or Siemens Healthcare), mainly including axial T1WI/volumetric interpolated breath-hold examination, T2WI, DWI, and MRCP. This study aimed to analyze axial T1WI, T2WI, and DWI findings. Table S1 lists the detailed parameters of the MRI sequences.

Table S1. Parameters of some MRI sequences.

| Parameters | Cohort 1 | | | Cohort 2 | | |
| --- | --- | --- | --- | --- | --- | --- |
|  | T1WI | T2WI | DWI | T1WI | T2WI | DWI |
| echo time (ms) | 1.44 | 70 | 52 | 1.20-2.25 | 71-80 | 49-58 |
| repetition time (ms) | 3.1 | 1610 | 934 | 3.5-5.5 | 1400-1650 | 880-950 |
| slice thickness (mm) | 3 | 7 | 7 | 3 | 7 | 7 |
| flip angle (degrees) | 10 | 90 | 90 | 8-12 | 90 | 90 |
| number of excitations | 1 | 2 | 4 | 1 | 2 | 4 |
| number of slices (slices) | 120 | 24 | 48 | 120 | 24 | 48 |
| b values (s/mm^2^) | - | - | 0 and 800 | - | - | 0, 800 or 1000 |

Axial T1 high resolution isotropic volume excitation sequence, T1WI; axial fat-suppressed turbo spin echo (TSE) T2-weighted imaging sequence, T2WI; axial diffusion weighted imaging, DWI.

**Supplementary material S****3**

**Manual segmentation**

First, tumor size (the maximum diameter on transverse images) was measured manually on higher b-value (b=800/1000 s/mm^2^) axial DWI with the maximum cross-sectional area of the tumor with reference to T1WI and T2WI images. Lesion diameter was measured by an experienced radiologist in cancer imaging using RadiAnt Digital Imaging and Communications in Medicine (DICOM) Viewer, 1.9.16 version software (Medixant, www.radiantviewer.com). Manual delineation of the tumor region of interest (ROI) was performed by 3 radiologists with >6 years of experience in abdominal imaging, using freely available software (ITKSNAP, http://www.itksnap.org). Any visual qualitative disagreement between 3 radiologists was resolved by consensus. Tumor ROI were delineated on each slice of T1WI, T2WI, and DWI, covering the whole tumor region, avoiding adjacent vessels, bile duct, and other tissues as far as possible.

**Supplementary material S4**

**Image** **preprocessing**

All MRI images of the patients were pre-processed using Python 3.7. In our training configuration, a resampling algorithm (image resample to [2, 2, 4] mm) was applied to standardize the image intensities, which could minimize the adverse influence caused by differences in MRI images from different scanners. Subsequently, a point was randomly selected from the entire image, which was used as the patch center to crop an image patch of [160, 160, 48] from the resampled image. Before the patch was fed into the model for training, an adaptive normalization method was applied to the patch. In the adaptive normalization method, the z-score with per image mean and standard calculated from the intensity range between the percentile of 0.1 and 99.9, was used to normalize the patch, and then the intensity value was clipped to [-1,1].

**Data augmentation**

To improve the generalization performance of the model, data augmentation was employed using Python 3.7 in our study, including random translation in the cropped image (0–5 mm), small random scaling (0.9–1.1), and small random rotation (-10° to +10°).

**Supplementary material S5**

In the SE module, one global average pooling following the bottle module squeezed the output feature maps channel-wise, aggregating each feature map channel to a value. The squeezed features were excited by two fully connected layers and produced a group of weights for per-channel modulation. A sigmoid layer was used to normalize the weights to [0, 1]. In addition, in each residual SE block, the SE-attentive feature maps were element-wise summed with the input feature maps to obtain the final output feature maps.

In this study, the proposed SE-VB-Net employed an anisotropic structure. Specifically, in the first down and last up blocks, the down-sampling and up-sampling operations were performed only on the x and y axes. In the rest of the down and up blocks, the down-sampling and up-sampling operations were performed on the x, y, and z axes. In other words, the kernel and stride sizes were set as 2×2×1 and 2×2×1 in the convolution/de-convolution module of the first down/last up block, respectively. In the other down/up blocks, the kernel and stride sizes were set to 2×2×2 and 2×2×2, respectively.

**Supplementary material S6**

The differences between the single-modality and combined network were as followed: (1) First, each modality image, including DWI, T1WI, and T2WI, were separately equipped with the input block and the first two down blocks; (2) Next, the following two down blocks, four up blocks, and one output block were shared for each modality; (3) a merge block following the first two separate down blocks was added to combine the output feature map of each modality. Before training the multi-modality model, T1WI and T2WI were registered in the DWI image space.

**Supplementary material S7**

**Loss functions**

The weights of the three loss functions (dice loss, focal loss, and soft dice loss) were equal. When calculating the loss function, the weights of the background and foreground classes were set to 1 and 10, respectively. The loss function is defined as follows:

$$L_{dice}=1-\frac{2\times\text{ }V_{P}\text{ × }V_{L}}{V_{P}\text{+ }V_{L}}$$

$$L_{focal}=-\alpha\left( 1-V_{p} \right)^{\gamma}\times V_{L}\log V_{p}-(1-\alpha){V_{p}}^{\gamma}(1-V_{L})\log{(1-V}_{p})$$

$$L_{soft dice}=1-\frac{2\times\text{ }V_{P}\text{ × }V_{L}}{{V_{P}}^{2}\text{+ }{V_{L}}^{2}}$$

where $V_{L}$ and $V_{P}$ denote the gold coronary mask and the predicted coronary mask.

**Supplementary material S8**

**Evaluation metrics for segmentation**

Manual delineations performed by a radiologist were used as the ground truth. To evaluate the accuracy of the segmentation algorithm, the results of the automatically segmented data were compared with those of the ground truth, using both volumetric and surface analysis statistics. Evaluation metrics, including the Dice similarity coefficient (DSC), 95th percentile of Hausdorff distance (HD95), average surface distance (ASD), and Jaccard similarity coefficient (JSC), were calculated using python3.7.

The DSC is defined as:

$$DSC=\frac{2\text{×}\text{ }V_{P}\text{ }\text{×}\text{ }V_{L}}{V_{P}\text{+ }V_{L}}$$

Here, $V_{P}$ and $V_{L}$ represent the predicted segmentation mask and ground truth annotated by radiologists, respectively.

The HD is defined as:

$$HD\left( S_{P}\text{ , }S_{L} \right)=\max_{p\in S_{P}} \min_{l\in S_{L}} d(p,l)$$

where $S_{P}$ and $S_{L}$ are the surface of $V_{p}$ and $V_{L}$, respectively.$d(p,l)$ is the Euclidean distance between two points *p* and *l* from $S_{P}$ and$\text{ }S_{L}$, respectively. HD is the maximum value of the surface distance, and thus HD95 is the 95 percentile value to remove the effect of outliers.

The ASD describing the average symmetric surface distance is defined as:

$$ASD(S_{P}\text{ , }S_{L})=\sum_{p\in S_{P}} {min}_{l\in S_{L}}d(p,l)/\left| S_{P} \right|$$

where $\left| S_{P} \right|$ indicates the total number of surface point.

The JSC is defined as:

$$JSC=\frac{V_{P}\text{×}\text{ }V_{L}}{\left| V_{P} \right|\text{+}\left| V_{L} \right|-\left| V_{P}\text{×}\text{ }V_{L} \right|}$$

The success rate of segmentation for each model, meaning its ability to detect coarse location of tumors. The case was regarded as a successful detection as long as its segmented DSC was greater than 0. The success rate is defined as:

$$Success rate=\frac{\sum_{i=1}^{n} D_{i}}{N}$$

$$D_{i}=\left\{ \begin{aligned} 0, if {DSC\_T}_{i}==0 \\ 1, other \end{aligned} \right.$$

where ${DSC\_T}_{i}$ is the calculated dice similarity score of the tumor $T_{i}$, $T_{i}\in T$, denoting $T$ is the tumor set, $T=\{T_{0}, T_{1},\cdots,T_{i},\cdots,T_{n-1}\}$, $i\in\{0, 1,\cdots,N-1\}$, N is the number of the tumor. Denoting $DSC\_T=\{{DSC\_T}_{0}, {DSC\_T}_{1}, \cdots,{DSC\_T}_{i},\cdots,{DSC\_T}_{n-1}\}$, $i\in\{0, 1,\cdots,N-1\}$, is the dice similarity score set of $T$.
